# Mechanical Properties of C_3_N Nanotubes from Molecular Dynamics Simulation Studies

**DOI:** 10.3390/nano10050894

**Published:** 2020-05-07

**Authors:** Azam Salmankhani, Zohre Karami, Amin Hamed Mashhadzadeh, Mohammad Reza Saeb, Vanessa Fierro, Alain Celzard

**Affiliations:** 1 Faculty of Mechanical Engineering, K. N. Toosi University of Technology, P.O. Box 1969764499 Tehran, Iran; azamsalmankhani@gmail.com; 2 Center of Excellence in Electrochemistry, School of Chemistry, College of Science, University of Tehran, P.O. Box 14155-6455 Tehran, Iran; zohrekarami.2013@yahoo.com; 3 Department of Mechanical Engineering, Azadshahr Branch, Islamic Azad University, P.O. Box 49617-89985 Azadshahr, Iran; 4 Department of Resin and Additives, Institute for Color Science and Technology, P.O. Box 16765-654 Tehran, Iran; mrsaeb2008@gmail.com; 5 Université de Lorraine, CNRS, IJL, 88000 Epinal, France; vanessa.fierro@univ-lorraine.fr

**Keywords:** C_3_N nanotubes, molecular dynamics, mechanical properties, nanobuds, defects

## Abstract

Although the properties of carbon nanotubes (CNTs) are very well-known and are still extensively studied, a thorough understanding of other carbon-based nanomaterials such as C_3_N nanotubes (C_3_NNTs) is still missing. In this article, we used molecular dynamics simulation to investigate the effects of parameters such as chirality, diameter, number of walls, and temperature on the mechanical properties of C_3_N nanotubes, C_3_N nanobuds, and C_3_NNTs with various kinds of defects. We also modeled and tested the corresponding CNTs to validate the results and understand how replacing one C atom of CNT by one N atom affects the properties. Our results demonstrate that the Young’s modulus of single-walled C_3_NNTs (SWC_3_NNTs) increased with diameter, irrespective of the chirality, and was higher in armchair SWC_3_NNTs than in zigzag ones, unlike double-walled C_3_NNTs. Besides, adding a second and then a third wall to SWC_3_NNTs significantly improved their properties. In contrast, the properties of C_3_N nanobuds produced by attaching an increasing number of C_60_ fullerenes gradually decreased. Moreover, considering C_3_NNTs with different types of defects revealed that two-atom vacancies resulted in the greatest reduction of all the properties studied, while Stone–Wales defects had the lowest effect on them.

## 1. Introduction

In recent decades, rapid progress has been made in the development of new classes of 2D and 1D materials, and these materials have entered almost all fields of science and technology. Graphene and CNTs as the most famous 2D and 1D nanostructures have been extensively used in a wide range of mechanical, electronic, optoelectronic, and medical applications [1,2,3,4,5,6,7,8]. The success of these materials relies on their extraordinary properties such as good electrical conductivity, excellent electron mobility, and high mechanical strength arising from their ultra-thin sp^2^ network along with high specific surface area [9,10,11].

The outstanding properties of carbon-based nanostructures have prompted researchers to focus on other types of carbon and non-carbon 1D, 2D, and 3D materials. For instance, metal oxide nanotubes and sheets including beryllium oxide (BeO), titanium oxide (TiO_2_), and zinc oxide (ZnO) [12,13,14,15], nitride-based sheets or tubular structures such as boron nitride (BN), gallium nitride (GaN), and aluminum nitride (AlN) [16,17,18], nanobuds [19,20,21], nanopeapods [22,23,24], etc., are worth citing. Among these structures, nitrides have great potential in various applications according to the reports provided by numerous studies. In a molecular dynamics (MD) study, Cong et al. studied the mechanical properties of BN-Al nanotubes/metal matrix nanocomposites. They found that the Young’s modulus, yield stress, and yield strain of the composites increased with the nanotubes diameter [25]. Ghorbanzadeh et al. [16] used the density functional theory (DFT) to examine the mechanical properties of single and multi-layer graphene-like sheets based on group III nitrides and showed that adding layers to the studied surfaces could increase the elastic modulus of AlN and BN sheets, but not GaN. Albooye et al. [26] used a MD method to consider the mechanical properties of boron nitride nanotubes (BNNTs) with point defects and doped by carbon atoms. They found that the concentration of vacancies could reduce their mechanical properties, and armchair BNNTs had a higher Young’s modulus than zigzag BNNTs. Besides, carbon-doped BNNTs had lower mechanical properties than pristine BNNTs in either zigzag or armchair configuration [26].

Another nitride-based nanostructure that has been much studied recently is 2D carbon nitride (C_3_N). This structure can form during the CNT synthesis This nanostructure has been the subject of many research works in recent years and has been used in a variety of applications such as energy storage, adsorption of CO_2_, NO_2_ and H_2_S, electrocatalysis, and photocatalysis [27,28,29,30,31,32,33,34,35]. In this regard, Sadeghzadeh used MD simulation to design C3N nanosheets and investigate the effect of holes on their tensile properties. He reported a considerable reduction in Young’s modulus, yield stress, and yield strain of the C_3_N nanosheets when the diameter of the holes increased [36]. Shirazi et al. [32] used MD simulation to study the mechanical behavior of C_3_N sheets under critical defects such as line cracks and notches, and found a lower mechanical strength of C_3_N when the crack line or notch diameter increased. A research based on density functional theory (DFT) was developed by Ma et al. to understand the ability of pristine and B-doped C_3_N to adsorb NO_2_. They demonstrated that pristine C_3_N has a high sensitivity to NO_2_ and that doping with B atoms considerably increases this sensitivity [34]. In another article, Faye et al. used pristine and defective C_3_N sheets for the adsorption of H_2_S and NH_3_. Their results revealed that the weak interaction between pristine C_3_N and NH_3_ or H_2_S in the gas phase was considerably strengthened once a single N or C defect was created on the surface of C_3_N [35]. The mechanical behavior of C_3_N sheets was investigated by Mortazavi in a combined DFT-MD research [30]. He reported 341 GPa for the elastic modulus from the DFT calculation, which was only 3% different from the MD estimates. Mortazavi also predicted a thermal conductivity of about 815 W m^-1^ K^-1^ [30] for free-standing C_3_N. He therefore proposed this nanostructure as a potential candidate for new applications such as reinforcement of polymer-based nanocomposites.

As discussed above, the structure and various properties of 2D C_3_N materials have been investigated in depth. However, less attention has been devoted to exploring the properties of C_3_N nanotubes (C_3_NNTs), i.e., 1D structures. To the best of the authors’ knowledge, no research has been developed so far to study the mechanical properties of pristine and defective C_3_N nanotubes as well as the 3D nanostructures that they can form, such as nanobuds. Such studies include chirality, tube dimension, number of walls, and the effect of temperature.

On the one hand, the structural defects that form during the process of nanotube synthesis play a crucial role in the mechanical performance of nanostructures, in particular at high temperatures. On the other hand, 3D nanostructures such as nanobuds have shown great potential for being widely used in various industrial applications. It is therefore necessary to study the new carbon nanostructures from the point of view of their mechanical properties, as this could open up a new perspective for future developments in nanotechnology.

The main goal of this article is therefore to use MD simulation to investigate the effect of chirality, tube diameter, number of walls, and temperature on elastic modulus, failure stress, and failure strain of flawless and defective C_3_NNTs, as well as C_3_N nanobuds. Since no research has been conducted so far to evaluate the properties of C_3_NNTs, we modeled and tested carbon nanotubes with the same chirality under the same loading condition to validate the results. The obtained results will be fully discussed and compared in the following sections.

## 2. Computational Method

In this study, MD simulation was performed with large-scale atomic/molecular massively parallel simulator (LAMMPS) for modeling the mechanical properties of C_3_NNTs under uniaxial tensile loading. In order to determine the interaction between carbon atoms, the optimized Tersoff potential presented by Lindsay and Broido [37] was used. Besides, the Tersoff potential parameters developed by Kinaci et al. [38] were used for determining the interaction between C and N atoms. C_3_NNTs with an approximate length of 50Å were modeled in armchair or zigzag chirality, and the obtained results were compared to the properties of carbon nanotubes (CNTs). MD simulations were carried out in two steps: first, the stress-free structures at the simulation temperature were obtained with the isothermal-isobaric (NPT) ensemble operating at 50 ps. Then, at an initial constant velocity, the simulation box was stretched along the loading axial direction. The simulations were carried out for nanotubes with various conditions such as chirality (zigzag or armchair), temperature (from 300 to 900 K), number of walls (from 1 to 3), and various defects (attachment of fullerene and point defects) to evaluate their impact on elastic modulus, failure stress, and failure strain.

## 3. Results

### 3.1. Geometrical Design

**Figure 1:**
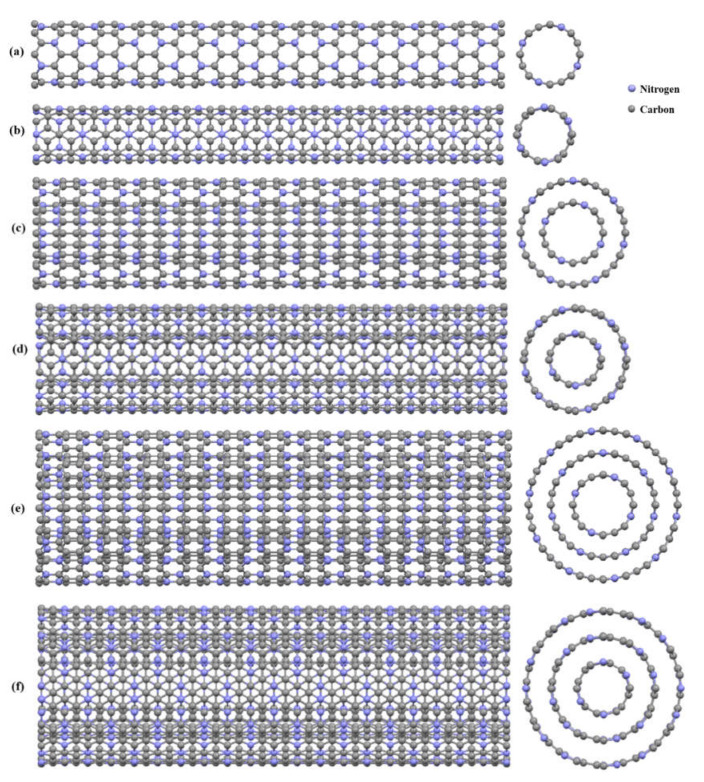
Schematic (side and cross-sectional) views of the modeled C_3_N nanotubes (C_3_NNTs): (**a**) (8,0) zigzag single-walled C_3_NNTs (SWC_3_NNT), (**b**) (4,4) armchair SWC_3_NNT, (**c**) ((8,0),(12,0)) zigzag double-walled (DWC_3_NNT), (**d**) ((4,4),(8,8)) armchair DWC_3_NNT, (**e**) ((8,0),(12,0),(20,0)) zigzag triple-walled (TWC_3_NNT), and (**f**) ((4,4),(8,8),(12,12)) armchair TWC_3_NNT.

**Figure 2:**
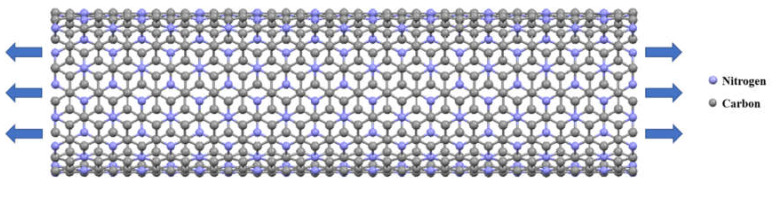
Schematic side view of an armchair SWC_3_NNT under uniaxial tensile loading.

As mentioned above, the main objective of this study was to determine the mechanical behavior of C_3_N nanotubes and nanobuds as a function of diameter, chirality, number of walls, and temperature as input variables. For that purpose, armchair and zigzag C_3_NNTs with one, two, and three walls were modeled using the MD simulation method. Schematic views of the modeled C_3_NNTs along with the data of their chirality and stoichiometry are given in Figure 1 and in Table 1. Next, all samples were subjected to uniaxial tensile loading, as shown in Figure 2, with a strain rate of 0.001 ps^−1^ at a constant temperature of 300 K to calculate Young’s modulus, failure stress, and failure strain from the corresponding stress–strain plots.

### 3.2. Mechanical Properties of Single-Walled C_3_NNTs

**Figure 3:**
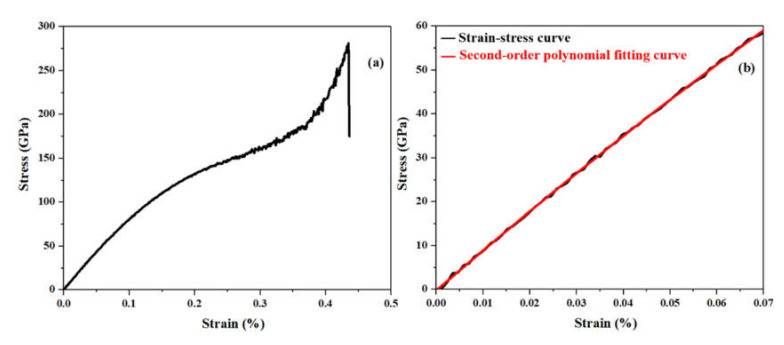
(**a**) Stress–strain curve for a (6,6) armchair C_3_NNT, and (**b**) second-order polynomial fit to the first part of the curve.

The Young’s modulus of each SWC_3_NNT was determined as follows. After plotting the stress–strain (*σ*–*ε*) curves, a second-order polynomial was fitted to the linear part of the curves, and the Young’s modulus was calculated using Equation (1) [39,40]:

(1) $$\sigma =\frac{\partial U}{\partial \varepsilon }=D\varepsilon^{2}+E\varepsilon +C$$

where *D*, *E*, and *C* are the third-order elastic modulus, the Young’s modulus, and the residual stress of the nanotubes, respectively. The stress–strain curve of a (6,6) armchair C_3_NNT at 300 K displayed in Figure 3a,b shows a zoom of the linear part used for the second-order polynomial fit.

**Figure 4:**
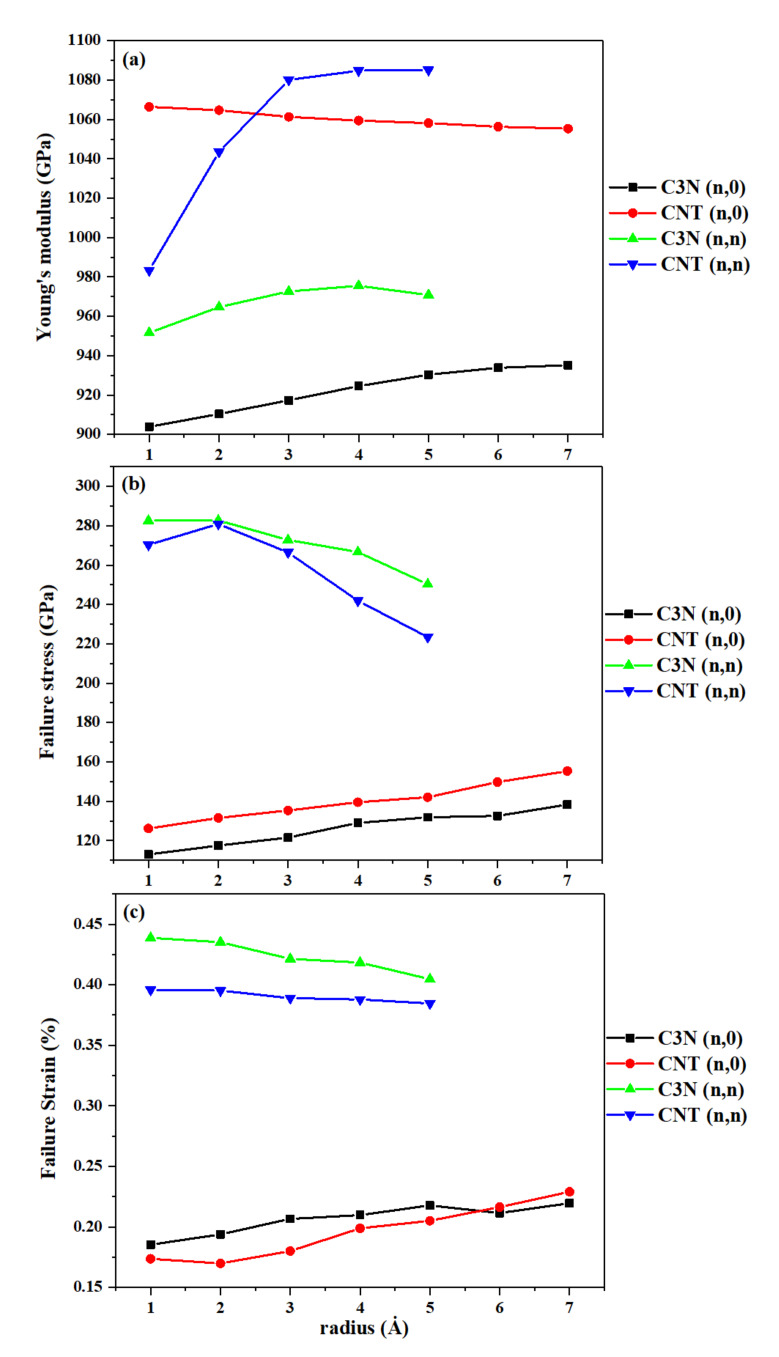
(**a**) Young’s modulus, (**b**) failure stress, and (**c**) failure strain of SWC_3_NNTs and SWCNTs under uniaxial tensile tests at 300 K, as a function of their radius.

According to Figure 3a, the highest stress obtained is observed at a strain of about 40%. Then, the stress drops to a considerably lower amplitude, therefore the coordinates of this point correspond to the values of failure stress and failure strain. We repeated these steps for all SWC_3_NNTs and then for SWCNTs to compare and validate our results. The plots of Young’s modulus, failure stress, and failure strain are displayed in Figure 4. This figure shows that the variation of all the studied properties as a function of the radius of the nanotubes is rather limited. Indeed, as seen in Figure 4a, by increasing the radius of the nanotubes, the Young’s modulus of armchair SWC_3_NNTs increases by about 24 GPa, passing from 951.6 GPa in structure (4,4) to 975.5 GPa in structure (10,10), and then drops to 970.8 GPa in structure (12,12). Similarly, an upward behavior is observed in the Young’s modulus of armchair CNTs, except for the thickest ones where the values tend to stabilize, rising from 983.3 GPa in structure (4,4) to 1085.4 GPa in structure (12,12).

We observed the same behavior by comparing the Young’s modulus of zigzag and armchair SWC_3_NNTs. The Young’s modulus of zigzag SWC_3_NNTs increases slightly, from 903.8 GPa in (8,0) to 935.2 GPa in (20,0), similar to the upward trend of armchair SWC_3_NNTs. A general comparison between the modulus of SWC_3_NNTs and SWCNTs reveals that the values obtained for SWC_3_NNTS are much lower than for the corresponding SWCNTs at any chirality or radius considered. The higher mechanical behavior of CNTs compared to C_3_NNTs is caused by the length of the C-C bond, which is about 1.445 Å in the ideal structure of CNTs, while the length of the C-N bond in C_3_Ns is about 1.468 Å. The shorter bond length between the elements of the nanostructures leads to higher mechanical properties as demonstrated by Ghorbanzadeh et al. [16]. As mentioned earlier, we modeled CNTs in the present paper to have the possibility of validating our simulation and obtaining results for C_3_NNTs. The Young’s modulus that we calculated for SWCNTs is around 1000 GPa, which is close to what has already been reported in previous theoretical articles, ranging from 0.5 to 1.5 TPa [41,42,43,44,45], as well as in experimental reports [46,47,48]. For instance, Treacy et al. reported a value of around 2 TPa for the elastic modulus of individual SWCNTs of different diameters and lengths [46], and Yu et al. reported an average modulus of 1002 GPa for 8 SWCNT ropes using atomic force microscopy (AFM) [48]. These results demonstrate the accuracy of our simulation and therefore also of our results for C_3_NNTs.

Furthermore, from Figure 4b,c, we can see that the failure stress and failure strain of zigzag SWC_3_NNTs and SWCNTs increase with the radius of the nanotubes, while those of armchair SWC_3_NNTs and SWCNTs decrease under the same conditions. The highest failure stress and failure strain, equal to 282.6 GPa and 0.44%, respectively, were found for armchair (6,6) and (4,4) C_3_NNTs, respectively. In an MD-case study, Shirazi et al. reported the ultimate tensile strength of armchair and zigzag C_3_N sheets at 300 K, equal to 128 GPa and 125 GPa, which are both lower than what we obtained for our strongest structures (250.21 GPa for armchair and 138.29 for zigzag) [32]. The results calculated for the mechanical properties of armchair and zigzag SWC_3_NNTs and SWCNTs are listed in Table 2 and Table 3.

**Figure 5:**
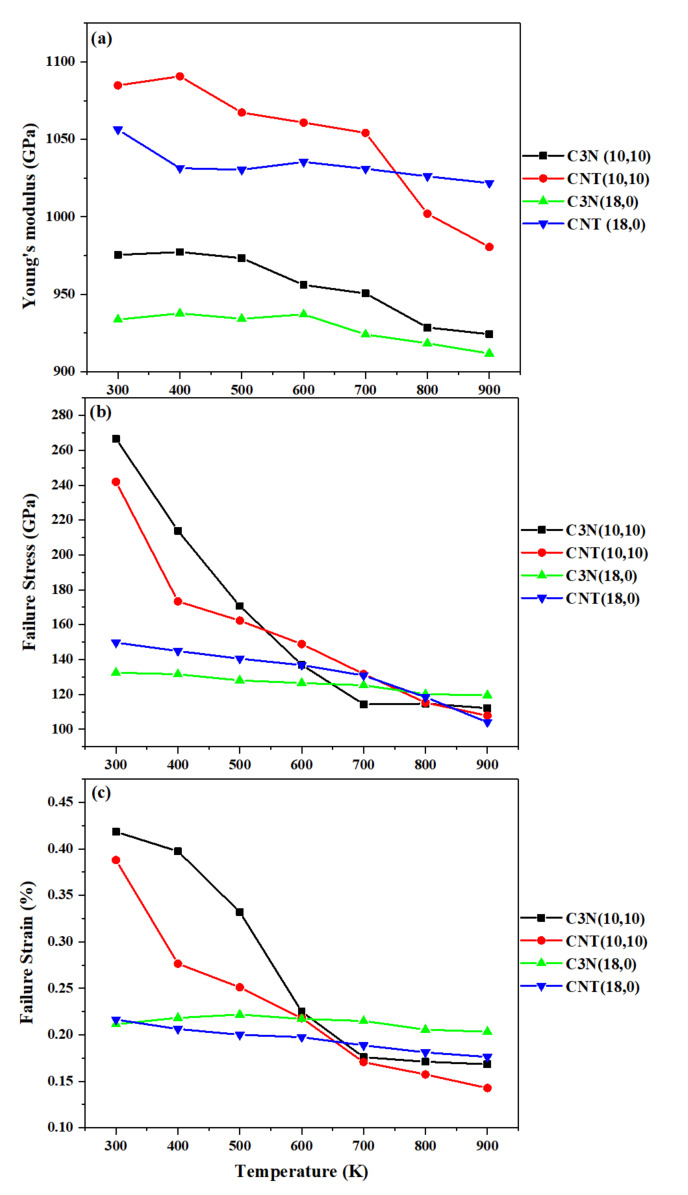
(**a**) Young’s modulus, (**b**) failure stress, and (**c**) failure strain of (10,10) and (18,0) SWC_3_NNTs and SWCNTs under uniaxial tensile tests, as a function of temperature.

To take the effect of temperature into account, (10,10) and (18,0) single-walled C_3_NNTs and the CNTs with the closest dimensions were modeled and subjected to a uniaxial tensile loading while the temperature increased from 300 to 900 K. The calculated results are shown in Figure 5.

All the aforementioned mechanical properties decreased with temperature, whether for CNTs or for C_3_NNTs. Thus, the highest values for all tested samples were obtained at 300 K and the lowest values at 900 K. The (10,10) armchair C_3_NNT had a higher elastic modulus at any temperature compared to the (18,0) zigzag structure, and the C_3_NNTs showed a lower modulus than the CNTs. The Young’s modulus of (18,0) and (10,10) C_3_NNTs were respectively 2% and 5% lower at 900 K than at 300 K. Likewise, the failure stress and failure strain of all the samples decreased when the temperature increased. However, although the failure stress of zigzag and armchair C_3_NNTs was lower than those of the corresponding CNTs at most temperatures, these structures generally failed at a higher strain rate than those of CNTs. The same observations were reported by Shirazi et al. [32]. They noted a total decrease of 36% for the stress at failure of C_3_N sheets at 900 K compared to 200 K, in accordance with our own finding.

### 3.3. Mechanical Properties of Double-Walled C_3_NNTs

**Figure 6:**
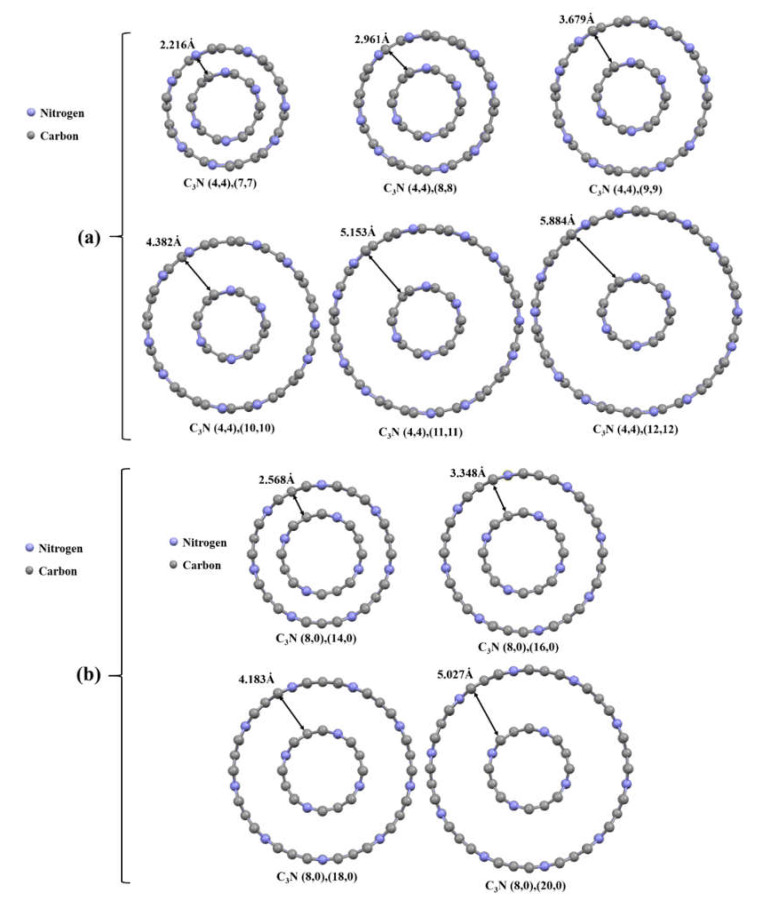
Cross-sectional view of the structure of studied C_3_NNTs nanotubes: (**a**) armchair DWC_3_NNTs, and (**b**) zigzag DWC_3_NNTs.

At this point, to probe the effect of adding walls to C_3_NNTs on their mechanical properties, we modeled and tested four zigzag and six armchair double-walled C_3_NNTs (DWC_3_NNTs) as well as the corresponding DWCNTs. It should be mentioned that there are some limitations to the modeling of multi-walled nanotubes, in particular regarding the interlayer distances. If the interlayer distance exceeds a certain value, no van der Waals interaction occurs between layers and the structure does not form. On the other hand, if the distance were less than a specific value, the structure would collapse because of instability. Therefore, to have an armchair and zigzag structures with the closest dimensions enabling reasonable comparisons to be made, we selected the distances according to the schematic views displayed in Figure 6. Thus, we could model stable structures with close and comparable dimensions.

**Figure 7:**
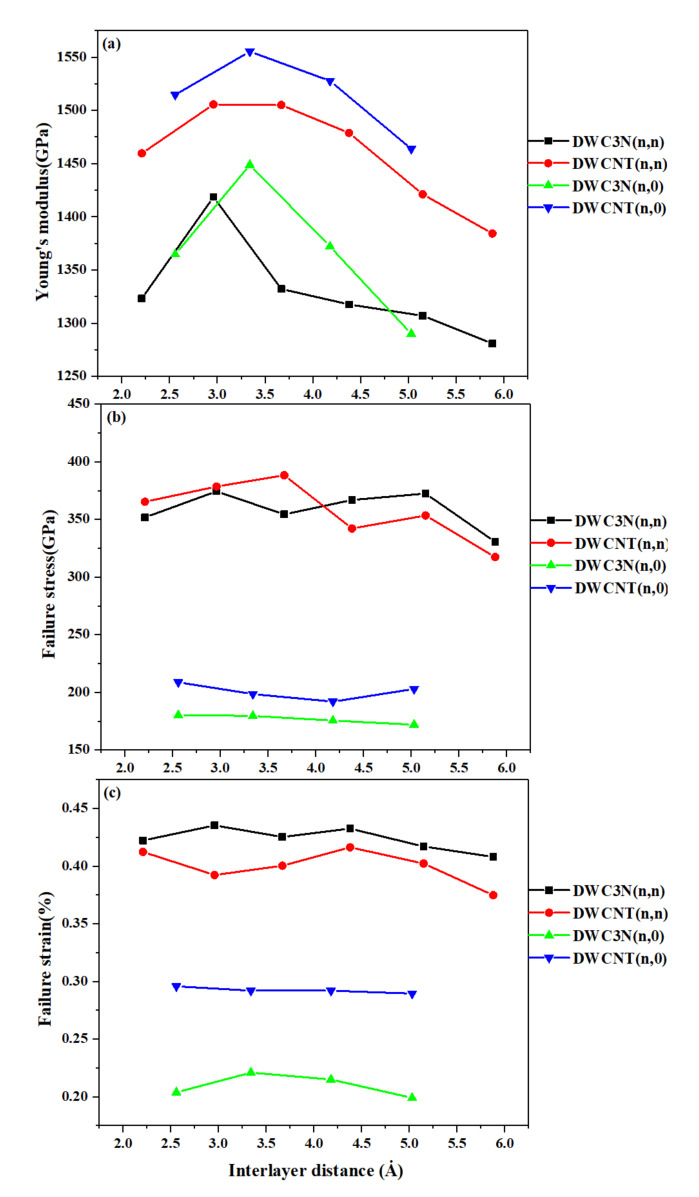
(**a**) Young’s modulus, (**b**) failure stress, and (**c**) failure strain of DWC_3_NNTs and DWCNTs under uniaxial tensile tests as a function of interlayer distance.

After testing all the modeled samples under uniaxial tensile loading at a constant temperature of 300 K, the mechanical properties were plotted in Figure 7. As seen in Figure 7a, by increasing the interlayer distance, the Young’s modulus of all samples first increased and then constantly decreased to slightly lower values. Unlike single-walled nanotubes, the Young’s modulus of zigzag DWC_3_NNTs and SWCNTs was higher than that of armchair DWC_3_NNTs of similar radius, while DWC_3_NNTs had a lower elastic modulus compared to DWCNTs, as already observed for single-walled nanotubes. The results of double-walled armchair and zigzag C_3_NNTs and CNTs are collected in Table 4 and Table 5, respectively. The highest values obtained for DWC_3_NNTs occurred in structures (8,0),(16,0) and (4,4),(8,8), 1448.7 GPa and 1418.6 GPa, respectively, and this property was about 10% lower in the weakest structure with respect to the strongest one, whatever the chirality.

Besides, making comparisons between the results of single and double-walled C_3_NNTs reveals that adding a wall to these nanotubes increased the elastic modulus so that DWC_3_NNTs have a considerably higher elastic modulus than SWC_3_NNTs. In addition, and just like SWCNTs, DWCNTs showed a higher Young’s modulus than DWC_3_NNTs regardless of the chirality. The Young’s modulus of DWCNTs was higher than that of SWCNTs, just like for C_3_NNTs. The highest Young’s modulus of DWCNTs found in the zigzag structure (8,0),(16,0), 1553.3 GPa, is close to that reported earlier [49,50], further supporting the accuracy of our simulation and the results calculated for DWC_3_NNTs.

Finally, considering Figure 7b,c, we found that failure stress and failure strain of zigzag DWC_3_NNTs were nearly two times lower than those of armchair DWC_3_NNTs having a close radius. No chirality showed a significant trend by increasing the radius, and a similar trend was observed for DWCNTs. We obtained the highest failure stress and failure strain of DWC_3_NNTs in the (4,4),(8,8) armchair: 374.4 GPa and 0.435%, respectively.

### 3.4. Mechanical Properties of Triple-Walled C_3_NNTs (TWC_3_NNTs)

**Figure 8:**
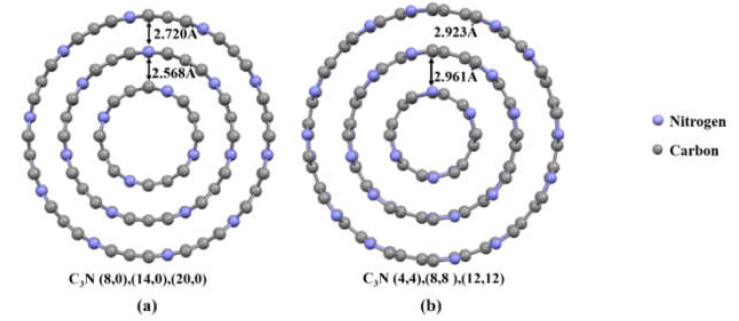
Schematic cross-sectional view of: (**a**) zigzag and (**b**) armchair TWC_3_NNTs.

In this section, we added one more wall to DWC_3_NNTs to compare the mechanical behavior of TWC_3_NNTs to that of SWC_3_NNTs and DWC_3_NNTs. For that purpose, we modeled and tested one zigzag and one armchair TWC_3_NNT of structures (8,0),(14,0),(20,0) and (4,4),(8,8),(12,12), respectively, and the corresponding TWCNTs. A schematic view of the modeled structures is displayed in Figure 8, and the obtained results are presented in Table 6.

By examining this table, we found a significant growth in the Young’s modulus of TWC_3_NNTs compared to double- or single-walled nanotubes, whether CNTs or C_3_NNTs, and regardless of chirality. The Young’s modulus of armchair TWC_3_NNT (1850.4 GPa) was higher than that of zigzag TWC_3_NNT (1760.7 GPa), unlike what we had obtained for DWC_3_NNTs. In addition, the Young’s modulus of TWC_3_NNTs was lower than that of TWCNTs with the same structure, as for single-walled C_3_NNTs and CNTs. Besides, the failure stresses of armchair TWC_3_NNT and TWCNTs were not only higher than for zigzag TWC_3_NNTs and TWCNTs, but also higher than for double- and single-walled nanotubes.

**Figure 9:**
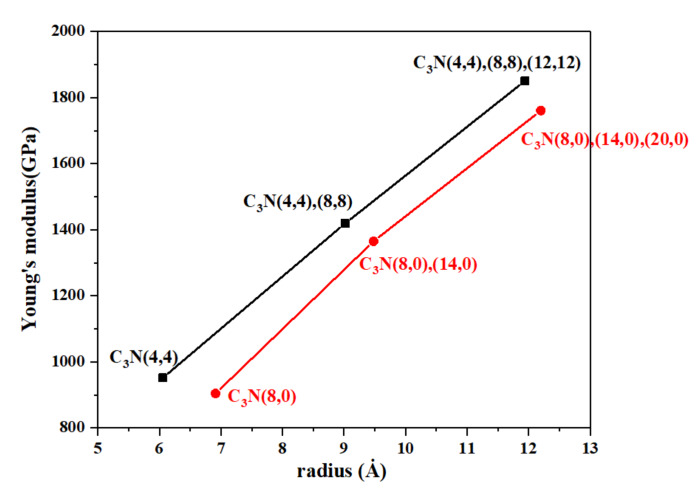
Young’s modulus as a function of nanotube radius for different types of single-, double-, and triple-walled chiral C_3_NNTs.

Moreover, we compared the mechanical properties of single-walled (4,4) and (8,0) C_3_NNTs with double- and triple-walled C_3_NNTs made up of these two basic structures in Figure 9. From this figure, it can be observed that the addition of walls to the single-walled nanotubes had a remarkable impact on the Young’s modulus of both C_3_NNTs and CNTs, regardless of the chirality. By increasing the radius following the addition of one then two more walls to the SWC_3_NNTs, the modulus of (4,4) single-wall armchair C_3_NNT increased by 32% and 48%, respectively, in structures (4,4),(8,8) and (4,4),(8,8),(12,12), respectively. Similarly, the modulus of (8,0),(14,0) and (8,0),(14,0),(20,0) zigzag structures were respectively 33% and 46% higher than that of the (8,0) SWC_3_NNT. The same behavior is observed in TWCNTs compared to their corresponding double and single-walled counterparts.

### 3.5. Mechanical Properties of C_3_N Nanobuds

**Figure 10:**
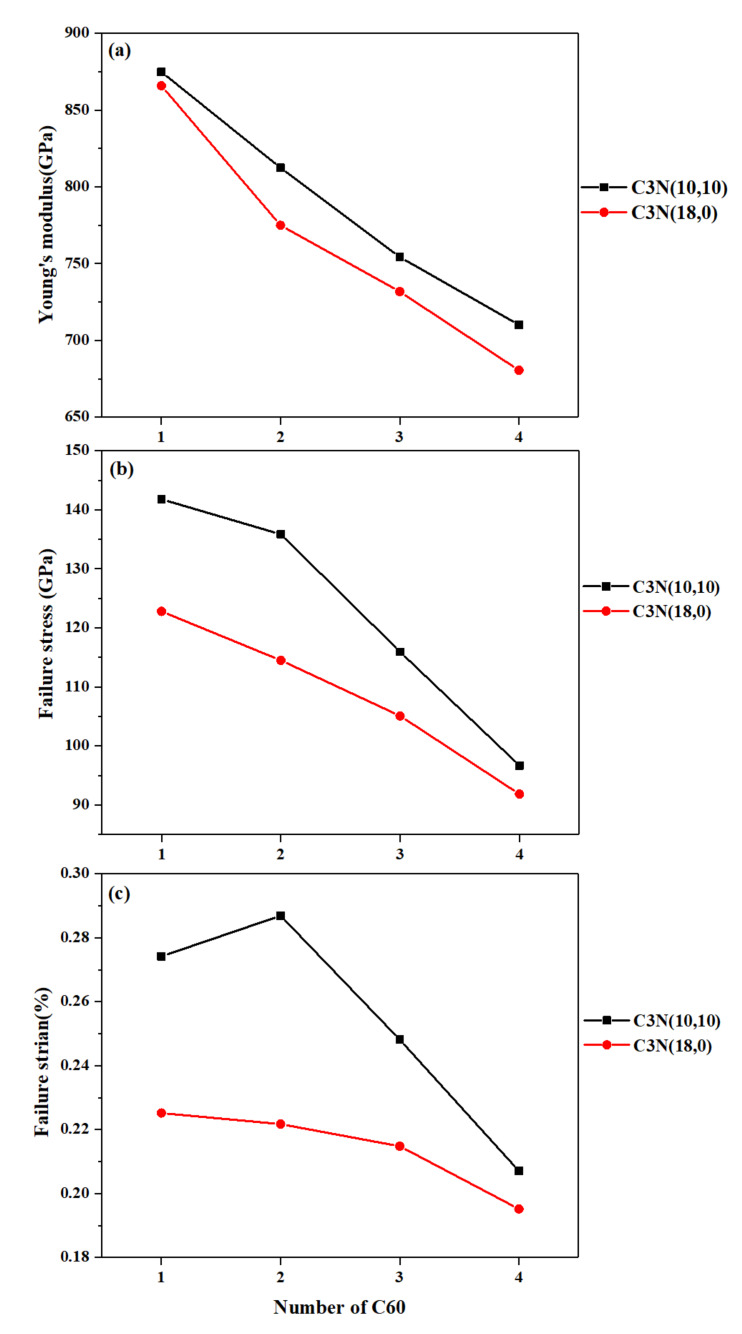
(**a**) Young’s modulus, (**b**) failure stress, and (**c**) failure strain of C_3_N nanobuds under uniaxial tensile tests at 300 K.

Nanobuds are 3D nanostructures that form when a fullerene or nanocage is randomly attached to the outer surface of nanotubes or graphenic structures in the synthesis process. The specific features of nanocages and fullerenes, including their porous shells and nanometric thickness, make them an appropriate choice for developing novel 3D nanostructures [51]. In the present work, we attached randomly one, two, three, and four C_60_ fullerene molecules to the outer surface of zigzag and armchair SWC_3_NNTs to form C_3_N nanobuds. One armchair and one zigzag C_3_NNTs with the closest dimension of structures (10,10) and (18,0) were modeled and tested. The plots of the mechanical properties of armchair and zigzag SWC_3_NNTs after attaching one, two, three, and four C_60_ to their surface are given in Figure 10.

Similar to what we had seen above for SWC_3_NNTs, the mechanical properties of armchair nanobuds were higher than zigzag nanobuds, and all the studied properties decreased constantly as the number of C_60_ increased. This could be due to first, the increase in the effective surface area of the nanobuds with the number of attached fullerenes, and second to the associated increase in stress concentration, a higher number of attached fullerenes implying a higher stress concentration. In addition, the properties of C_3_N nanobuds were lower than for simple SWC_3_NNTs, either in zigzag or armchair structures. The highest elastic modulus was calculated for structures (10,10)-1C_60_: 874.5GPa, and (18,0)-1C_60_: 865.8 GPa, i.e., was 10% and 7% lower than (10,10) and (18,0) SWC_3_NNTs, respectively. With four C_60_ attached to armchair and zigzag nanobuds, the modulus was reduced by almost 20% compared to those with only one C_60_. The same kind of results have been reported by other studies on other types of nanobuds. Mashhadzadeh et al. in their DFT-based research, reported that the Young’s modulus of graphene-like BeO reduced considerably by increasing the number of attached nanocages [20]. Ghorbanzadeh et al. used DFT calculations to compare the mechanical properties of simple CNTs with CNT nanobuds. They found a reduction in Young’s modulus of armchair and zigzag CNTs after attaching a C_60_ molecule to their surface. The values obtained for the mechanical properties of C_3_N nanobuds are presented in Table 7.

**Figure 11:**
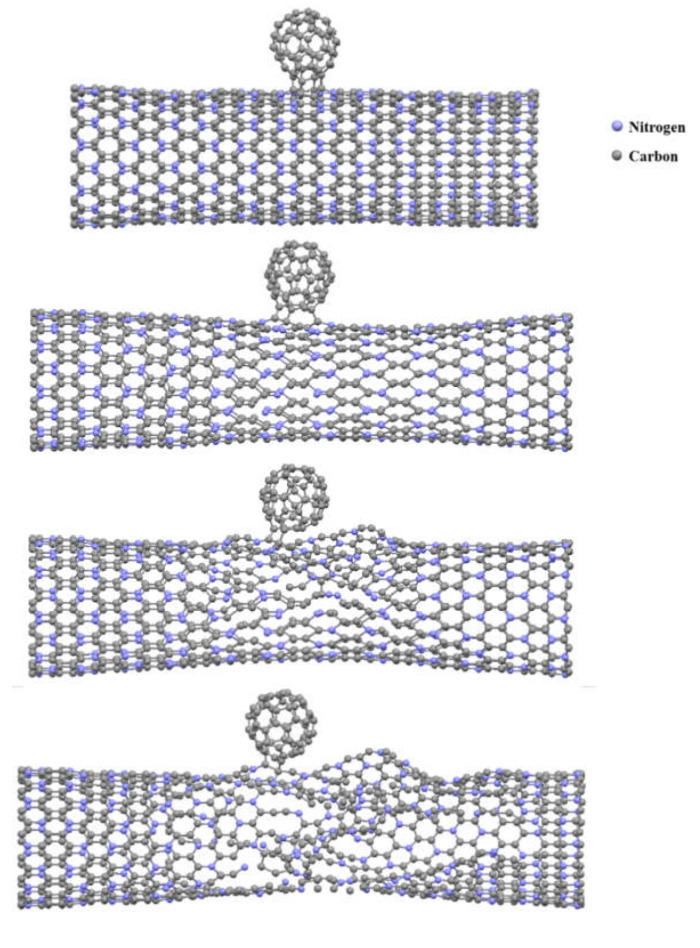
Snapshot of the failure process of a (18,0)-1C_60_ C_3_N nanobud.

Figure 11 shows a snapshot of the failure process of a (18,0)-1C_60_ SWC_3_NNT. It can be seen that, as expected, the failure started around the region where the fullerene was placed on the nanotube surface. This is due to the higher stress concentration existing around this region, which facilitates the formation and propagation of cracks.

### 3.6. Mechanical Properties of Defective C_3_NNTs

**Figure 12:**
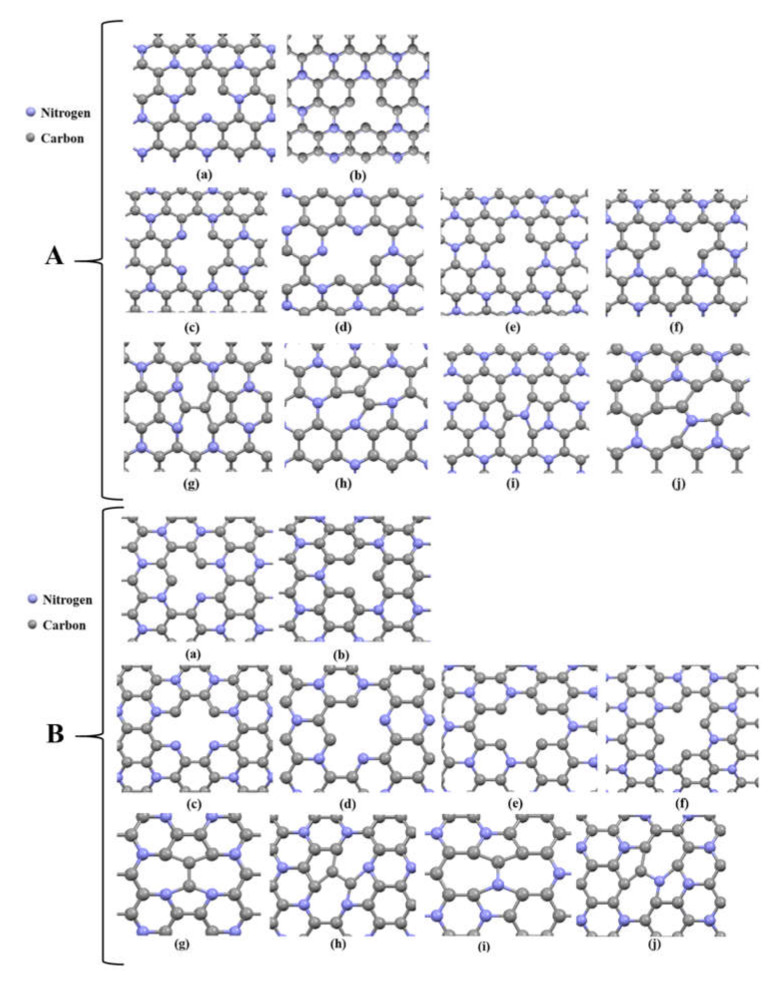
(**A**) Embedded defects in (10,10) armchair C_3_NNT: (a) one carbon atom vacancy, (b) one nitrogen atom vacancy, (c) and (d) two carbon atoms vacancy, (e) and (f) one-carbon and one-nitrogen vacancy, (g) and (i) Stone-wales type 1, (h) and (j) Stone-wales type 2. (**B**) Same as (**A**) but for (18,0) armchair C_3_NNT.

In the end, we examined the effect of point defects on the mechanical properties of SWC_3_NNTs. (10,10) armchair and (18,0) C_3_NNTs were selected and we modeled the defective samples with one and two vacancies as well as Stone–Wales defects. All the designed samples are presented in Figure 12, where the two types of Stone–Wales defects can be seen. Type one (STW-1) forms when a horizontal C-N (or C-C) bond rotates 90 degrees, and type 2 (STW-2) forms once a skewed C-N (or C-C) bond rotates 90 degrees.

**Figure 13:**
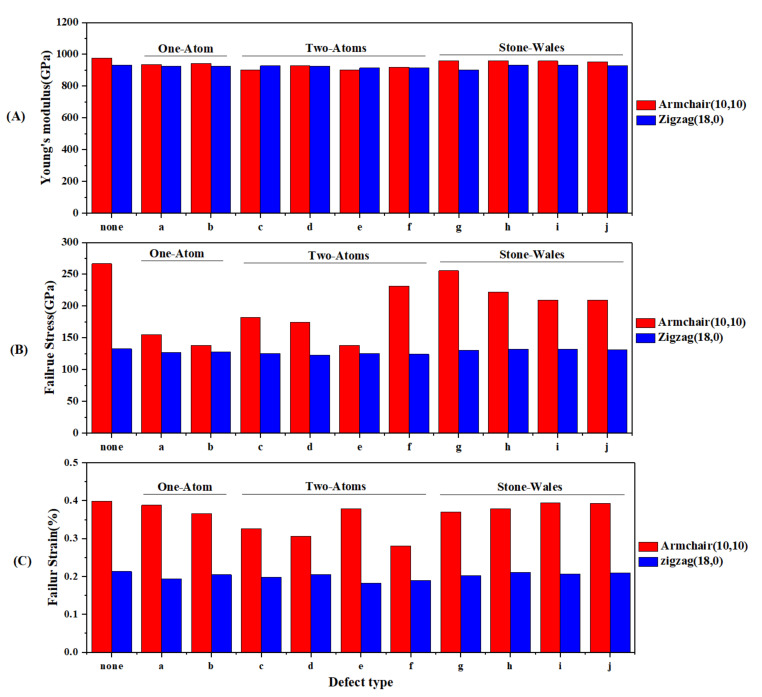
(**a**) Young’s modulus, (**b**) failure stress, and (**c**) failure strain of defective C_3_NNTs at 300 K

After having implemented tensile tests at a constant temperature of 300 K and at constant strain rate ε of 10^8^ s^−1^, the results are presented as bar graph in Figure 13. According to this figure, creating defects on the surface of armchair and zigzag C_3_NNTs resulted in a reduction of all mechanical properties compared to pristine SWC_3_NNTs. The Young’s modulus of different defected zigzag and armchair C_3_NNTs are close to each other (with higher values for most types of armchair), with very little differences and no significant trend. However, the failure stress and failure strain of defective armchair C_3_NNTs are considerably higher than the corresponding zigzag C_3_NNTs. The results obtained for each property are collected in Table 8 for each type of defect.

This table shows that the highest reduction in all properties occurred when two atoms were removed from the surface of zigzag and armchair SWC_3_NNTs. In contrast, defects of types STW-1 and STW-2 resulted in the lowest reduction in properties compared to pristine SWC_3_NNTs. The lowest Young’s modulus, failure stress, and failure strain, 900.3 GPa, 122.68 GPa, and 0.182%, respectively, were about 7%, 9%, and 15% lower than for the corresponding pristine SWC_3_NNTs. These values corresponded to two-atom vacancy C_3_NNTs, including armchair structure defect type c (2-C), zigzag structure defect type d (2-C), and zigzag structure defect type e (1-C, 1-N), respectively. The same kind of results have been reported by previous researches. Shirazi et al. provided the same results for C_3_N nanosheets with crack-type defects [32]. They showed that increasing the crack length could significantly decrease the mechanical response of the defective sheets. However, Sadeghzadeh et al. observed different results for their C_3_N sheet depending on the vacancy concentration [31]. They demonstrated that defective C_3_N had higher elastic modulus and failure strain than the defect-free sheets, which is different from the findings of Shirazi’s and ours. In another article, an adverse effect of point defects on the mechanical properties of graphene-like ZnO structures was reported by Ghorbanzade et al. [15]. In a MD-based study, Albooye et al. also reported that increasing the number of missing atoms reduced the Young’s modulus of defective BNNTs so that the highest modulus was obtained in pristine structures, and the lowest modulus was observed in those containing three-atom vacancies [26]. In another MD studies, Gupta et al. investigated Young’s modulus, failure stress, and failure strain of hybrid single-layer graphene; they reported a reduction in the behavior of all properties with respect to non-defective graphene monolayers by imposing Stone–Wales and nanopore defects [52].

**Figure 14:**
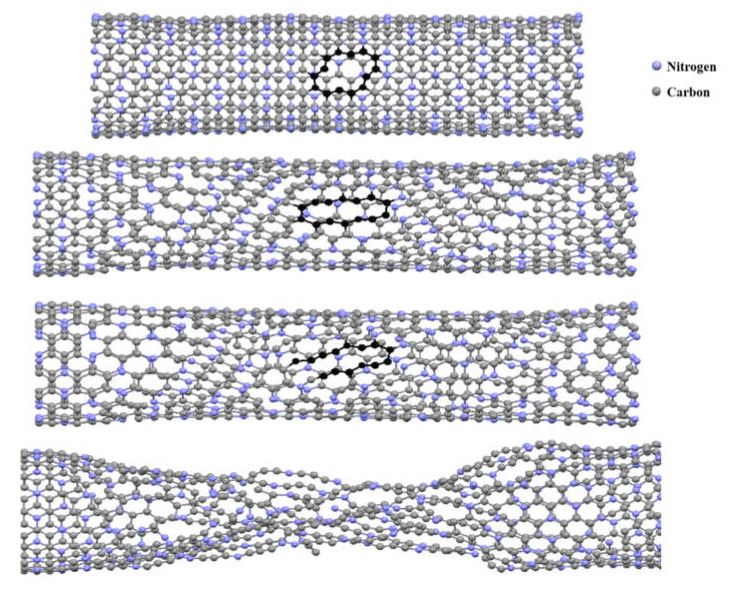
Snapshot of the failure process of a (10,10) armchair C_3_NNT with a two-atom vacancy defect.

Furthermore, a snapshot of the failure process of a (10,10) armchair SWC_3_NNT including a two-atom vacancy defect is presented in Figure 14. Similar to what happens in nanobuds, the failure of defective C_3_NNT begins from the defective region due to its higher stress concentration, and then the cracks propagate until the complete rupture of the structure.

## 4. Conclusions

Molecular dynamics simulation has been used in the present work to determine the impact of parameters such as nanotube radius, number of walls, number of attached fullerenes, defect type, and temperature on the mechanical properties of single-, double-, triple-walled C_3_NNTs, defective C_3_NNTs, and C_3_N nanobuds. The results revealed that adding walls to SWC_3_NNTs improved the mechanical behavior whether in zigzag or armchair chirality. The properties of armchair nanotubes were higher than zigzag ones, and were negatively affected by the temperature. Besides, the elastic properties were improved by increasing the number of walls. The highest moduli of armchair and zigzag SWC_3_NNTs, 975.5 GPa and 935.1 GPa, respectively, were obtained in structures (10,10) and (20,0), respectively. Those of armchair and zigzag (10,10) and (18,0) TWC_3_NNTs were 1850.4 GPa and 1760.7 GPa, respectively, i.e., nearly 47% higher than the corresponding SWC_3_NNTs. Furthermore, the failure properties generally decreased by increasing the radius of the nanotubes. The Young’s modulus, failure stress, and failure strain of armchair SWC_3_NNTs and DWC_3_NNTs were higher than zigzag ones, and these properties increased by adding walls to the nanotubes.

Additionally, we considered C_3_N nanobuds and found that their mechanical properties were lower than pristine C_3_NNTs. This was especially the case when more fullerenes were attached to them, so that the Young’s modulus of a nanobud with four C_60_ was 20% less than for a nanobud with only one C_60_, whatever the chirality. Finally, we imposed vacancies and Stone–Wales defects to SWC_3_NNTs. Our results demonstrated that two-atom vacancies and Stone–Wales defects resulted in the highest and smallest drop, respectively, in Young’s modulus, failure stress, and failure strain, whatever the chirality. The minimum Young’s modulus of armchair and zigzag C_3_NNTs occurred with defects type c (2C removed): 900.3 GPa, and type e (1C and 1N removed): 914.6 GPa. Overall, the outcomes of the present article offer a new perspective for developing new carbon-based nanotubes for broad industrial applications.

## Figures and Tables

**Table 1:** Stoichiometry of single-walled C_3_NNTs (SWC_3_NNTs) and multi-walled (double-walled and triple-walled) C_3_NNTs (MWC_3_NNTs).

Chirality of SWC_3_NNTs	Number of Atoms	Chirality of MWC_3_NNTs	Number of Atoms
(4,4)	328	(4,4),(7,7)	902
(6,6)	492	(4,4),(8,8)	984
(8,8)	656	(4,4),(9,9)	1066
(10,10)	820	(4,4),(10,10)	1148
(12,12)	984	(4,4),(11,11)	1230
(8,0)	384	(4,4),(12,12)	1312
(10,0)	480	(8,0),(14,0)	1056
(12,0)	576	(8,0)(16,0)	1152
(14,0)	672	(8,0),(18,0)	1248
(16,0)	768	(8,0),(20,0)	1344
(18,0)	864	(4,4),(8,8),(12,12)	1968
(20,0)	960	(8,0),(14,0),(20,0)	2016

**Table 2:** Mechanical properties of armchair SWC_3_NNTs and SWCNTs under uniaxial tensile tests at 300 K.

Nanotube	Properties	Chirality				
		(4,4)	(6,6)	(8,8)	(10,10)	(12,12)
**C_3_NNTs**	Young’s Modulus (GPa)	951.6	964.7	972.6	975.5	970.8
	Failure Stress (GPa)	282.54	282.64	272.73	266.62	250.21
	Failure Strain (%)	0.415	0.398	0.402	0.399	0.412
**CNTs**	Young’s Modulus (GPa)	983.3	1043.5	1080.1	1084.8	1085.1
	Failure Stress (GPa)	270.25	280.83	266.41	241.85	223.41
	Failure Strain (%)	0.395	0.395	0.388	0.387	0.384

**Table 3:** Same as Table 2, but for zigzag SWC_3_NNTs and SWCNTs.

Nanotube	Properties	Chirality						
		(8,0)	(10,0)	(12,0)	(14,0)	(16,0)	(18,0)	(20,0)
**C_3_NNTs**	Young’s Modulus (GPa)	903.8	910.4	917.3	924.5	930.3	933.8	935.1
	Failure Strain (%)	112.94	117.48	121.54	128.96	131.84	132.52	138.29
	Failure Strain (%)	0.290	0.253	0.212	0.213	0.205	0.213	0.218
**CNTs**	Young’s Modulus (GPa)	1066.4	1064.7	1061.3	1059.4	1058.1	1056.3	1055.4
	Failure Strain (%)	126.15	131.52	135.32	139.46	142.03	149.71	155.41
	Failure Strain (%)	0.173	0.169	0.180	0.198	0.205	0.216	0.229

**Table 4:** Mechanical properties of armchair DWC_3_NNTs and DWCNTs under uniaxial tensile tests at 300 K.

Nanotube	Properties	Chirality					
		(4,4),(7,7)	(4,4),(8,8)	(4,4),(9,9)	(4,4), (10,10)	(4,4),(11,11)	(4,4),(12,12)
**C_3_NNTs**	Young’s Modulus (GPa)	1323.2	1418.6	1332.2	1317.6	1307	1280.8
	Failure Stress (GPa)	351.68	374.36	354.29	366.86	372.42	330.45
	Failure Strain (%)	0.422	0.435	0.425	0.432	0.417	0.408
**CNTs**	Young’s Modulus (GPa)	1459.6	1505.6	1505	1478.7	1421.1	1384.3
	Failure Stress (GPa)	365.26	378.55	388.28	342.13	353.40	317.32
	Failure Strain (%)	0.412	0.392	0.400	0.416	0.402	0.374

**Table 5:** Same as Table 4, but for zigzag DWC_3_NNTs and DWCNTs.

Nanotube	Properties		Chirality		
		(8,0),(14,0)	(8,0),(16,0)	(8,0),(18,0)	(8,0),(20,0)
**C_3_NNTs**	Young’s Modulus (GPa)	1364.7	1448.7	1372.1	1289.9
	Failure Stress (GPa)	180.24	179.54	175.42	171.91
	Failure Strain (%)	0.203	0.220	0.214	0.198
**CNTs**	Young’s Modulus (GPa)	1514.7	1553.3	1527.7	1463.8
	Failure Stress (GPa)	208.72	198.50	191.93	202.86
	Failure Strain (%)	0.295	0.291	0.291	0.289

**Table 6:** Mechanical properties of zigzag and armchair TWC_3_NNTs and TWCNTs under uniaxial tensile tests at 300 K.

Properties	Chirality			
	(4,4),(8,8),(12,12)	(8,0),(14,0),(20,0)	(4,4),(8,8),(12,12)	(8,0),(14,0),(20,0)
Young’s Modulus (GPa)	1850.4	1760.7	2050.1	1886.9
Failure Stress (GPa)	510.25	388.30	500.01	355.18
Failure Strain (%)	0.419	0.423	0.414	0.490

**Table 7:** Mechanical properties of C_3_N nanobuds under uniaxial tensile tests at 300 K.

Chirality	Properties	Number of Attached Fullerenes			
		1	2	3	4
armchair	Young’s Modulus (GPa)	874.8	812.4	754.3	710.1
	Failure Stress (GPa)	141.77	135.84	115.92	96.67
	Failure Strain (%)	0.274	0.286	0.248	0.207
zigzag	Young’s Modulus (GPa)	865.8	774.9	731.8	680.5
	Failure Stress (GPa)	122.7	114.5	105.08	91.82
	Failure Strain (%)	0.225	0.221	0.214	0.195

**Table 8:** Mechanical properties of defective SWC_3_NNTs.

Chirality	Properties	Defect Type										
		none	a	b	c	d	e	f	g	h	i	j
armchair	Young’s Modulus (GPa)	975.5	935.3	943.1	900.3	927.4	901.4	918.5	959.6	959.3	957.5	952.1
	Failure Stress (GPa)	266.62	155.18	138.30	182.47	174.23	138.07	231.59	266.62	221.81	209.37	209.35
	Failure Strain (%)	0.399	0.388	0.365	0.326	0.306	0.378	0.280	0.370	0.379	0.395	0.393
zigzag	Young’s modulus (GPa)	933.8	925.1	924.6	927.9	924.4	914.6	916.7	902.24	933	932.1	929.3
	Failure Stress (GPa)	135.52	126.74	127.41	124.87	122.68	124.93	123.98	130.29	132.09	132.11	131.50
	Failure Strain (%)	0.213	0.193	0.204	0.197	0.204	0.182	0.189	0.202	0.211	0.205	0.209
